# Effect of Combination Therapy Using Hypothermia and Granulocyte Colony-Stimulating Factor in a Rat Transient Middle Cerebral Artery Occlusion Model

**DOI:** 10.6091/ibj.13852.2014

**Published:** 2014-10

**Authors:** Laya Ghahari, Manouchehr Safari, Mohamad Taghi Joghataei, Mehdi Mehdizadeh, Mansoureh Soleimani

**Affiliations:** 1 *Dept. of Anatomy, Medical School, Iran University of Medical Science, Tehran, Iran;*; 2 * Dept. of Anatomy, Medical School, AJA University of Medical Sciences, Tehran, Iran; *; 3 *Dept. of Anatomy, Medical School, Semnan University of Medical Science, Semnan, Iran;*; 4 * Cellular and Molecular Research Center, Faculty of Advanced Technology in Medicine, Dept. of Anatomy, Iran University of Medical Sciences, Tehran, Iran*

**Keywords:** Granulocyte colony-stimulating factor (G-CSF), Rats, Brain ischemia, Hypothermia

## Abstract

**Background:** Stroke is the third leading cause of death. Hypothermia has been recognized as an effective method in reducing brain injury. In this study, we assessed the effects of granulocyte colony-stimulating factor (G-CSF) as a neuroprotective agent and mild hypothermia on mortality, behavioral function, infarct volume, and brain edema in Wistar rats. **Methods:** Forty male rats were used in five groups (eight rats in each group): control, hypothermy, G-CSF, combination hypothermy + CSF, and sham. Rats were anesthetized by injection of chloral hydrate (400 mg/kg) intraperitoneally. Transient cerebral ischemia was induced by 60-min intraluminal occlusion of left middle cerebral artery. Hypothermia, initiated at the time of reperfusion and G-CSF was started one hour after reperfusion at a dose of 15 mg/kg subcutaneously. The motor behavior was measured using Garcia’s index and animals were assigned for the assessments of infarction, brain swelling, and mortality rate.** Results:** The mortality was 38.46% (control group) and reduced in other groups. Neurological deficit score of control group (40.31 ± 1.56) was significantly lower than in treatment groups. The total cerebral infarct volume of treatment group was significantly lower than control group (43.96 ± 44.05 mm^3^). Treatment with hypothermy plus G-CSF (2.69 ± 0.24%) could significantly reduce brain swelling volume than other treatment groups. **Conclusion: **Our major finding is that mild hypothermic treatment plus G-CSF significantly reduced mortality rate and edema and improved neurological function. The results suggest that the combination of hypothermia and G-CSF is more effectively than other treatment groups being used alone.

## INTRODUCTION

Stroke is a leading cause of human death and disability in worldwide [1], and inflammation appears to play an important role in the pathogenesis of ischemic stroke [2]. Ischemic stroke results from a transient or permanent reduction in cerebral blood flow that is restricted to the territory of a major brain artery. Reduction in blood flow, in most cases, is caused by the occlusion of a cerebral artery [3]. Cytotoxic edema is a significant clinical problem that can be developed in response to a large middle cerebral artery (MCA) occlusion and has been associated with approximately 80% mortality rate. Cerebral vascular occlusion initiates a sequence of events involving cell swelling [4] and a series of biochemical events with histopathological consequences, which, if not blocked, leads to neuronal death [5].

Hypothermia has been recognized as an effective method in reducing brain injury caused by a variety of neurological insults [6]. The neuroprotective effects of mild hypothermia have been well documented in experimental models [7]. It has been reported that hypothermia prevents cell death by multiple pathways [-]. The hematopoietic factor granulocyte-colony stimulating factor (G-CSF) effectively reduces infarct size and improves functional outcome after various types of experimental stroke [10, 11]. A few studies have demonstrated that the members of hematopoietic cytokine family (For example, G-CSF) have neuroprotective effects and/or support neurogenesis [12, 13] and bind to their respective receptors on the membranes of neurons and glial cells in the CNS to stimulate intracellular signaling pathways [13].

So far, there are no reports in the literature about the effect of post-ischemic hypothermia and G-CSF simultaneously.

In the present study, we assessed the treatment effects of G-CSF and mild hypothermia on mortality, behavioral function, infarct volume, and brain edema in Wistar rats.

## MATERIALS AND METHODS

***Animals.*** A total of 56 male Wistar rats (280-330 g) supplied by Animal Lab of Iran University (Tehran) were used. The rats were kept in standard cages in a temperature (22°C), humidity (40–60%), and light period (07.00–19.00 hour) controlled environment with free access to food and water. Of 56 rats, 16 were withdrawn from the study for various reasons (mortality and anesthesia). The numbers reported in the results refer to the number of animals that survived after the surgery. The other 40 rats were randomly divided into five groups:

1. Control ischemic group (n = 8) that submitted to 60 minutes of ischemia [5, 6, 13, 14] and 24 hours of reperfusion and then rats kept for seven days.
2. Hypothermia group (n = 8) that submitted to 60 minutes of ischemia and 24 hours of reperfusion. Post-ischemic hypothermia (33.5-35°C) [5] was initiated at the time of reperfusion for 30 minutes, and then rats kept for seven days.
3. G-CSF group (n = 8) that submitted to 60 minutes of ischemia and 24 hours of reperfusion, and then G-CSF (F. Hoffmann-La Roche, Switzerland) treatment was started one hour after reperfusion at a dose of 15 mg/kg subcutaneously and continued daily for seven days [15].
4. Hypothermia and G-CSF group (n = 8) that submitted to 60 minutes of ischemia and 24 hours of reperfusion. Then, procedures in groups 2 and 3 were carried out together. Next, the rats were kept for seven days.
5. Sham group (n = 8) simulation of the surgical procedure, with the introduction of the obstructer, but without occlusion of the MCA.

***Induction of transient focal cerebral ischemia. *** Rats were anesthetized by injection of chloral hydrate (400 mg/kg) intraperitoneally. Body temperature was intermittently recorded using a rectal thermometer (chicco, Australia) and kept between 37 and 38°C using a 220 V lamp next to the animal. Post-ischemic hypothermia was obtained by placing the animals in a container containing ice on neck and temporal region immediately after surgery [5]. The left cervical vessels were exposed through a ventral midline incision under a surgical microscope (Olympus Szx12). Obstruction of the MCA was performed by inserting a 4-0 nylon suture in the left internal carotid artery via external carotid artery until it reach the anterior cerebral artery [16, 17]. This method placed the tip of the suture at the origin of the anterior cerebral artery, thereby occluding the MCA [17]. To evaluate changes in neurological function associated with ischemia, the rats were subjected to a variety of somatosensory and motor tests before and after surgery. All testing was performed from 9 to 11 AM by the same investigator. The somatosensory and motor behavior indexes of the rats were measured using Garcia’s index on postoperative days. Six items were measured and the total score ranged from 3 to 18; the higher the score, the better the motor performance. Items 1–4 (spontaneous activity, symmetry of movements, symmetry of the forelimbs, and climbing the wall of wire cage) measured motor performance, and items 5 and 6 (reaction to touch on and response to vibrissae touch) measured sensory function [14].

**Fig. 1:**
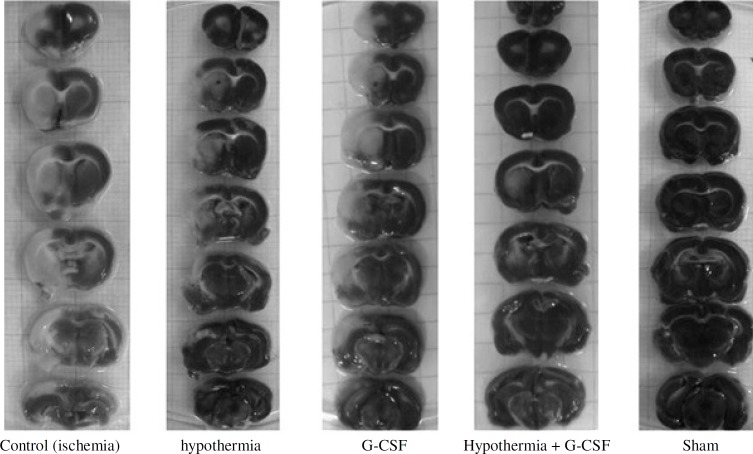
Representative of brain slices stained with triphenyltetrazolium chloride in different groups. Ischemic regions are colored white (light) and non-ischemic region are red (dark). G-CSF, granulocyte colony-stimulating factor

***Assessment of cerebral infarction and brain swelling.*** For defining the size of cerebral infarction, the animals were euthanized under ketamine (44 mg/kg, i.p.) and xylazine (13 mg/kg, i.p.) anesthesia. The brain was then rapidly removed, cut into 2-mm thick coronal sections by using brain matrix, stained with 2,3,5-triphenyltetrazolium chloride at room temperature for 30 minutes and then fixed in 10% buffered formalin [17, 18]. The infarct area on each slice was determined by using a Canon camera (IXUS 1000 HS, 10×), and the infarct areas (mm^2^) were calculated to obtain the infarct volumes per brain (mm^3^) by Image J 1.46r software (NIH, Wayne Rasband, USA) (Fig. 1). Infarct volumes were expressed as a percentage of the contralateral hemisphere volume by using an “indirect method” (area of intact contralateral [right] hemisphere minus area of intact regions of the ipsilateral [left] hemisphere) to compensate for edema formation in the ipsilateral hemisphere [17, 19]. Percentage of brain swelling was derived from volumetric growth of the ischemic hemisphere in comparison to the intact one as percentage of brain swelling (edema) = [(right hemisphere’s volume/left hemisphere’s volume)-1] ×100 [20].

***Statistical analyses.*** All data were analyzed by SPSS 16.0. ANOVA was used as appropriate for comparison among different groups followed by post hoc test (Tukey) for multiple comparisons. All data are expressed as mean ± SEM, and *P*≤0.05 was considered statistically significant.

## RESULTS

***Mortalities.*** The mortality of groups after ischemia was 38.46% (ischemic group), 11.11% (G-CSF group) and zero (other groups). Mortality was decreased in G-CSF group and was not observed in other treatment groups.

**Fig. 2:**
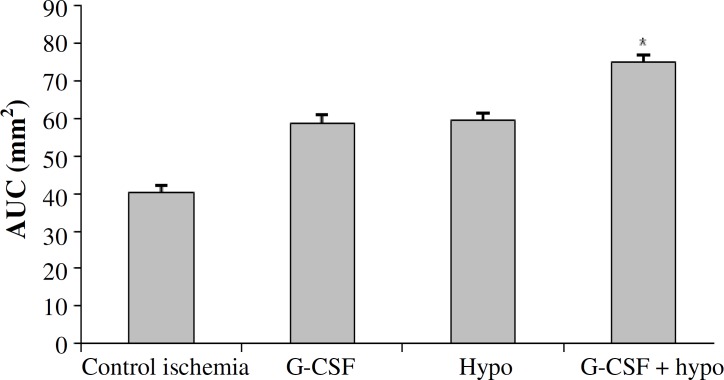
The comparison of neurological deficit scores (AUC) in different groups. *Significant difference versus all groups (*P*≤0.05). Granulocyte colony-stimulating factor (G-CSF); hypo, hypothermy

***Neurological deficit score.*** To determine the neurological deficit score of ischemic rat, as described in somatosensory and motor test (Garcia's index), the area under a curve was used (Fig. 2). Neurological deficit score of control ischemia group (40.31 ± 1.56) was significantly lower than sham (90 ± 0) and treatment groups. However, neurological deficit score of hypothermia group (59.43 ± 1) was not significantly different from G-CSF (58.81 ± 1), but it was significantly lower than hypothermia + GCSF 74.87 ± 2 and sham group.

**Fig. 3:**
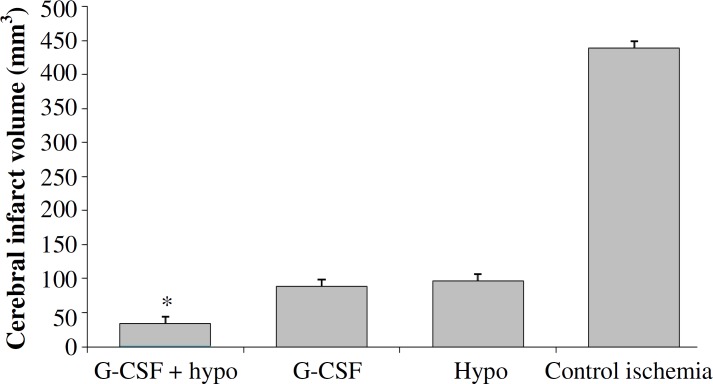
Total cerebral infarct volumes in ischemic and treatment groups. *Significant difference versus all groups (*P*≤0.05). Granulocyte colony-stimulating factor (G-CSF); hypo, hypothermy

***Cerebral infarct volumes.*** The appearance of white color combined with dark red color areas in the left hemispheres of ischemic rats (Fig. 1) indicated that left MCA occlusion was induced without affecting the right hemispheres. Quantitative comparisons of total cerebral infarction volumes have been shown in Figure 3. The rats in sham group had no cerebral infarction. The total cerebral infarct volume of treatment group (2, 3, and 4) was significantly lower than control ischemia group (4396 ± 44.05 mm^3^). However, all of the treatment groups could reduce the size of infarction especially when the rats were treated with hypothermy plus G-CSF (335 ± 18.23mm^3^).

**Fig. 4:**
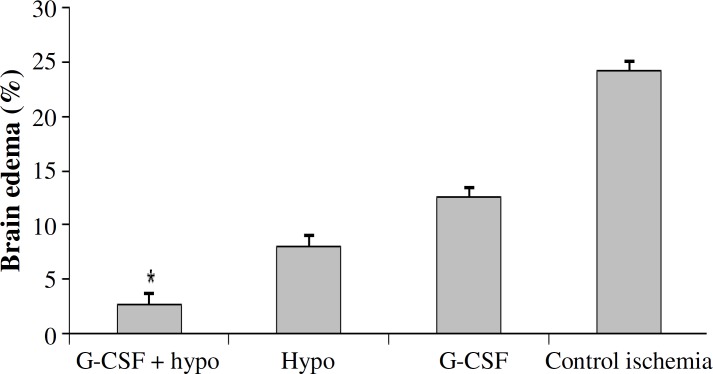
Brain edema in experimental groups. *Significant difference versus all groups (*P*≤0.05). Granulocyte colony-stimulating factor (G-CSF); hypo, hypothermy

***Brain swelling volumes (BSV, brain edema).*** BSV was caused by ischemic edema as shown in Figure 4. BSV in control ischemic group (24.13 ± 2.14%) were significantly higher than that in other groups. Treatment with hypothermy (8 ± 0.41%) and G-CSF alone (12.49 ± 0.72%) were significantly reduced BSV. Treatment with hypothermy plus G-CSF (2.69 ± 0.24%) could significantly reduce BSV as compared to other treatment groups. This decrease was not significantly different from sham group. 

## DISCUSSION

In this study, we used mild hypothermia because fewer side effect [21] and reduce edema and as known as neuroprotective agents. It has been generally accepted that reducing the body temperature to 33-34°C is neuroprotective against cerebral ischemic insults without causing many side effects [-]. Although the reduction in neurological and functional deficit and apoptosis was no longer observed after delaying hypothermia for two hours [25], a recent study suggested that it should be initiated in the exact moment of reperfusion, and that one hour of duration is enough [26]. Based on these recent concepts, in the present study, we used post-ischemic hypothermia started with reperfusion. However, we found significant difference between animals of the post-ischemic hypothermia group and control ischemic group regarding extension and volume of the ischemia. In a previous study, infarct volume was reduced by 22.4% in mild hypothermia [27], which is in agreement with our result.

Brain edema is life-threatening complication of cerebral ischemia, and one of the major determinants of patient’s survival after the first few hours of stroke [28]. Our results showed that hypothermy has a correlation with edema and mortality rate. However, in an older study, the reduced edema in the presence of normal blood pressure besides other mechanisms probably improves the re-establishment of microcirculation of the ischemic region and helps the repair of damages carried out during ischemia [28].

A previous study reported that G-CSF treatment in aged rats led to a reduced mortality, an improved functional recovery of motor function [10]. In addition to enhanced recovery of post stroke motor and cognitive function, G-CSF treatment clearly reduced mortality in aged rats after focal cerebral ischemia [10]. These results corroborate the findings of earlier studies in young mice and rats, in which a single intravenous treatment with G-CSF reduced mortality after MCA occlusion [10, 29]. In general, this survival enhancing capacity of G-CSF may be interpreted as a strong treatment effect in aged rats. This study confirms that short hypothermic treatment can be protective when it is applied with G-CSF.

Hypothermia has been considered the most effective resource to reduce brain injury caused by ischemia in experimental studies [26], and our results showed that mild hypothermy reduces mortality rate and infarct size. Similar results on infarct volume have been also reported [1, 30]. Meta-analysis of a previous study in young rats suggests that G-CSF treatment can reduce infarct size [12] . In the present study, an infarct reducing effect of G-CSF treatment was observed. G-CSF, in particular, has been shown to improve neurological function after various types of focal cerebral ischemia [30]. These results are consistent with our data.

Male animals were chosen in this study, because there is evidence that females have neuroprotection, due to sex specific hormones. Adult animals were used, because younger rats, usually weighing less than 280 g, have more developed collateral vessels, providing greater resistance to infarction.

Morphometric analysis of area and volume of focal cerebral ischemia represents an objective way to estimate the extent of ischemic injury, and it is commonly used for measuring the efficacy of neuroprotective agents [31].

Our study demonstrated that hypothermy and G-CSF could reduce mortality rate. We observed that treatment with hypothermy plus G-CSF reduced edema more effectively than other treatments (hypothermy or G-CSF alone).

Our major finding is that short hypothermic treatment plus G-CSF significantly reduces mortality rate, and edema as well as improves neurological function between this group and control ischemia group. The results suggest that the combination of hypothermia and G-CSF is more effectively than other treatment groups in our study, and this combination is important in improving motor function. Thus, hypothermy may provide the beneficial effect for the recovery of stroke patients by decreasing edema, and G-CSF may protect neurons from apoptosis.

## References

[1] Choi KE, Hall CL, Sun JM, Wei L, Mohamad O, Dix TA (2012 Jul). A novel stroke therapy of pharmacologically induced hypothermia after focal cerebral ischemia in mice. FASEB J.

[2] Jin R, Yang G, Li G (2010 May). Inflammatory mechanisms in ischemic stroke: role of inflammatory cells. J Leukoc Biol.

[3] Dirnagl U, Iadecola C, Moskowitz MA (1999 Sep). Pathobiology of ischaemic stroke: an integrated view. Trends Neurosci.

[4] Zador Z, Stiver S, Wang V, Manley GT Role of aquaporin-4 in cerebral edema and stroke. Handb Exp Pharmacol.

[5] Dezena RA, Colli BO, Carlotti Junior CG, Tirapelli LF (2012 Aug). Pre, intra and post-ischemic hypothermic neuroprotection in temporary focal cerebral ischemia in rats: morphometric analysis. Arq Neuropsiquiatr.

[6] Krieger DW, Yenari MA (2004). Therapeutic hypothermia for acute ischemic stroke: what do laboratory studies teach us?. Stroke.

[7] Liu L, Yenari MA (2007 Jan). Therapeutic hypothermia: neuroprotective mechanisms. Front Biosci.

[8] Alva N, Carbonell T, Palomeque J (2010 May). Hypothermic protection in an acute hypoxia model in rats: Acid-base and oxidant/antioxidant profiles. Resuscitation.

[9] Askalan R, Wang C, Shi H, Armstrong E, Yager JY (2011). The effect of postischemic hypothermia on apoptotic cell death in the neonatal rat brain. Dev Neurosci.

[10] Popa-Wagner A, Stöcker K, Balseanu AT, Rogalewski A, Diederich K, Minnerup J (2010 May). Effects of granulocyte-colony stimulating factor after stroke in aged rats. Stroke.

[11] England TJ, Gibson CL, Bath PM (2009 Dec). Granulocyte-colony stimulating factor in experimental stroke and its effects on infarct size and functional outcome: A systematic review. Brain Res Rev.

[12] Minnerup J, Heidrich J, Wellmann J, Rogalewski A, Schneider A, Schäbitz WR (2008 Jun). Meta-analysis of the efficacy of granulocyte-colony stimulating factor in animal models of focal cerebral ischemia. Stroke.

[13] Kong T, Choi JK, Park H, Choi BH, Snyder BJ, Bukhari S (2009 Jul). Reduction in programmed cell death and improvement in functional outcome of transient focal cerebral ischemia after administration of granulocyte-macrophage colony-stimulating factor in rats Laboratory investigation. J Neurosurg..

[14] Kim G, Kim E The effects of antecedent exercise on motor function recovery and brain-derived neurotrophic factor expression after focal cerebral ischemia in rats. J Phys Ther Sci.

[15] Buga AM, Bălşeanu A, Popa-Wagner A, Mogoantă L (2009). Strategies to improve post-stroke behavioral recovery in aged subjects. Rom J Morphol Embryol.

[16] Sicard KM, Fisher M (2009 Nov). Animal models of focal brain ischemia. Exp Transl Stroke Med.

[17] Mokudai T, Ayoub IA, Sakakibara Y, Lee EJ, Ogilvy CS, Maynard KI (2000 Jul). Delayed treatment with nicotinamide (Vitamin B(3)) improves neurological outcome and reduces infarct volume after transient focal cerebral ischemia in Wistar rats. Stroke.

[18] Kramer M, Dang J, Baertling F, Denecke B, Clarner T, Kirsch C et al (2010 Mar). TTC staining of damaged brain areas after MCA occlusion in the rat does not constrict quantitative gene and protein analyses. J Neurosci Methods.

[19] Shmonin A, Melnikova E, Galagudza M, Vlasov T (2012 Dec). Characteristics of cerebral ischemia in major rat stroke models of middle cerebral artery ligation through craniectomy. Int J Stroke.

[20] Strbian D, Karjalainen-Lindsberg ML, Tatlisumak T, Lindsberg PJ Cerebral mast cells regulate early ischemic brain swelling and neutrophil accumulation. J Cereb Blood Flow Metab.

[21] van der Worp HB, Sena ES, Donnan GA, Howells DW, Macleod MR (2007 Dec). Hypothermia in animal models of acute ischaemic stroke: a systematic review and meta-analysis. Brain.

[22] Maier CM, Ahern Kv, Cheng ML, Lee JE, Yenari MA, Steinberg GK (1998 Oct). Optimal depth and duration of mild hypothermia in a focal model of transient cerebral ischemia: effects on neurologic outcome, infarct size, apoptosis, and inflammation. Stroke.

[23] Tang XN, Liu L, Yenari MA (2009 Mar). Combination therapy with hypothermia for treatment of cerebral ischemia. J Neurotrauma.

[24] Kollmar R, Schwab S (2009 Mar). Hypothermia in focal ischemia: implications of experiments and experience. J Neurotrauma.

[25] Zgavc T, Ceulemans AG, Hachimi-Idrissi S, Kooijman R, Sarre S, Michotte Y (2012). The neuroprotective effect of post ischemic brief mild hypothermic treatment correlates with apoptosis, but not with gliosis in endothelin-1 treated rats. BMC Neurosci.

[26] Ginsberg MD (2008 Sep). Neuroprotection for ischemic stroke: past, present and future. Neuropharmacology.

[27] Schaller B, Graf R (2003 Dec). Hypothermia and stroke: the pathophysiological background. Pathophysiology.

[28] Panahpour H, Dehghani GA (2012). Attenuation of focal cerebral ischemic injury following post-ischemic inhibition of angiotensin converting enzyme (ACE) activity in normotensive rat. Iran Biomed J.

[29] Gibson CL, Bath PM, Murphy SP (2005 Apr). G-CSF reduces infarct volume and improves functional outcome after transient focal cerebral ischemia in mice. J Cereb Blood Flow Metab.

[30] Minnerup J, Sevimli S, Schäbitz WR (2009 Oct). Granulocyte-colony stimulating factor for stroke treatment: mechanisms of action and efficacy in preclinical studies. Exp Transl Stroke Med.

[31] Westermaier T, Zausinger S, Baethmann A, Steiger HJ, Schmid-Elsaesser R (2000 Nov). No additional neuroprotection provided by barbiturate-induced burst suppression under mild hypothermic conditions in rats subjected to reversible focal ischemia. J Neurosurg.

